# Antimicrobial potential of consolidation polymers loaded with biological copper nanoparticles

**DOI:** 10.1186/s12866-016-0766-8

**Published:** 2016-07-11

**Authors:** Ashraf M. M. Essa, Mohamed K. Khallaf

**Affiliations:** Botany Department, Faculty of Science, Fayoum University, Fayoum, Egypt; Biology Department, Faculty of Science, Jazan University, Jazan, Saudi Arabia; Conservation Department, Faculty of Archaeology, Fayoum University, Fayoum, Egypt

**Keywords:** Copper nanoparticles, Green synthesis, Antibacterial, Antifungal, Functionalized polymers

## Abstract

**Background:**

Biodeterioration of historic monuments and stone works by microorganisms takes place as a result of biofilm production and secretion of organic compounds that negatively affect on the stone matrix.

**Methods:**

Copper nanoparticles (CuNPs) were prepared biologically using the headspace gases generated by the bacterial culture *Escherichia coli* Z1. The antimicrobial activity of CuNPs was evaluated against the bacterial strains *Bacillus subtilis*, *Micrococcus luteus, Streptomyces parvulus, Escherichia coli*, *Pseudomonas aeruginosa* as well as some fungal strains *Aspergillus niger, Aspergillus flavus, Penicillium chrysogenum, Fusarium solani* and *Alternaria solani*.

**Results:**

Biological CuNPs demonstrated antibacterial and antifungal activities higher than those of the untreated copper sulfate. At the same time, limestone and sandstone blocks treated with consolidation polymers functionalized with CuNPs recorded apparent antimicrobial activity against *E. coli*, *S. parvulus* and *B. subtilis* in addition to an improvement in the physical and mechanical characters of the treated stones. Furthermore, the elemental composition of CuNPs was elucidated using electron dispersive x-ray system connected with the scanning electron microscope.

**Conclusion:**

Consolidation polymers impregnated with CuNPs could be used to restrain microbial deterioration in addition to the refinement of physico-mechanical behavior of the historic stones.

## Background

Nanoparticles demonstrate vast array of properties such as optical, electrical, catalytic, magnetic and biological activities which are diverged from those of the original constituents [1, 2]. The emergence of nanotechnology in the last decade offers occasions for exploring the antimicrobial effect of metal nanoparticles. Some of the biological properties of nanoparticles of various metals have been explored by assaying their antimicrobial susceptibilities. It has been reported that nanoparticles of Ag, Zn, Cu and Au exhibit a wide spectrum of antimicrobial activity against different bacterial [3–5] and fungal species [6–9].

Copper nanoparticles were reported to have antimicrobial activity against wide spectrum of bacteria including *Micrococcus luteus, Klebsiella pneumoniae, Escherichia coli, Pseudomonas aeruginosa, Staphylococcus aureus, Bacillus subtilis,* [10–12]. Moreover, CuNPs have been shown to suppress vegetative growth of some fungal species such as *Aspergillus flavus, Aspergillus niger, Alternaria alternata, Fusarium solani, Penicillium chrysogenum and Candida albicans* [13, 14].

Elevated levels of heavy metals represent a potential stimulus for metal tolerant bacteria that regularly possess specific metal resistance mechanisms. One of these mechanisms is the intracellular or extracellular transformation of metal ions into insoluble metal particles [15, 16]. The intracellular approach consists of transporting metal ions into the bacterial cell where they are transformed into nanoparticles while the extracellular process involves the trapping of metal ions on the cell surface as metal nanoparticles [17, 18]. Moreover, bacteria can release certain metabolites into their microenvironment that can transform metal ions into less soluble metal particles [19]. Synthesis of metal nanoparticles through bacteria is supported by the fact that the generated particles are environmentally safe and have elevated chemical reactivity [20].

Microorganisms can initiate and accelerate some geochemical reactions leading to biodeterioration of historic monuments [21]. The biodeterioration of archeological stones occurs as a consequence of the intrusion of microorganisms into the components of the mineral lattice [22]. The capability of microbial cells to inhabit stone surface was attributed to numerous aspects such as mineral composition, surface texture, moisture content, pH and nutrient accessibility [23]. In order to protect the archeological artifacts against microorganisms, different inorganic materials such as titanium dioxide and Ag-doped titanium dioxide have been used as antifouling agent by dispersing them in consolidation polymers. [24, 25]. Thus the aim of the present study was to investigate the antimicrobial potentiality of CuNPs synthesized biologically by the bacterial strain *Escherichia coli* Z1 and its application for the fortification of archaeological stones against microbial inhabitation.

## Methods

### Consolidation polymers and stone samples

In this study, two consolidation polymers were utilized. The first is Primal AC33 polymer (AC; Dow Chemical Co., USA) which comprises of methylacrylate and ethylmethacrylate. The other one is silicon polymer (S; Wacker BS 1001, Wacker Chemei AG, Germany) that is consisting of silane/siloxane emulsion. Sandstone and limestone samples were used in this study. The physical and mechanical properties of the tested stones including bulk density, water absorption, porosity, compressive strength and tensile strength were characterized before and after treating them with the functionalized polymers according to Essa and Khallaf [19].

### Preparation of the Cu-particles

A stock solution of copper sulfate was prepared by dissolving 200 mg of CuSO_4_ in 200 mL deionized distilled H_2_O. Different concentrations of CuSO_4_ solutions (50, 100, 150, 200, 250 μg/mL) were prepared from stock solution. One hundred milliliter of each concentration was exposed to the culture biogases of the bacterial strain *Escherichia coli* Z1 [26] for 60 min in aerobic bioreactor at 30 °C as described by Essa et al. [27]. Bacterial growth was monitored by measuring the optical density at 600 nm. The produced colloidal solution of each concentration was subjected for ultra-speed centrifugation at 100,000 rpm for 30 min. The collected Cu-particles was suspended in 10 mL deionized distilled H_2_O and re-centrifuged at 100,000 rpm for 30 min. This step was repeated three times and the collected Cu-particles were suspended in 1 mL dd H_2_O to assay the antimicrobial activities. Another set of the Cu-particles was suspended in the consolidation polymers at 150 μg/mL for stone treatments.

### Antibacterial activity of the Cu-particles

The antibacterial activity of copper particles was assayed against *Bacillus subtilis*, *Micrococcus luteus, Streptomyces parvulus, Escherichia coli* Z1 and *Pseudomonas aeruginosa*. Twenty five milliliter of nutrient broth containing various doses of Cu-particles (50, 100, 150, 200 and 250 μg/mL) were inoculated with 1 mL of a fresh culture of each bacterial strain (O.D = 0.6). After incubation for 48 h at 30 °C, the bacterial growth was monitored spectrophotometrically by measuring the optical density at 600 nm. At the same time, the antibacterial properties of the Cu-particles and CuSO_4_ were measured using the modified agar well diffusion method of Perez et al., [28]. Nutrient agar plates were inoculated with the different bacterial strains. Once the agar was solidified, it was punched with 8 mm diameter wells and filled with 25 μL of 100 μL/mL CuSO_4_ and Cu-particles. The experiment was repeated three times with three replicates for each treatment and diameters of the inhibition zones were measured after 24 h incubation at 30 °C. Streptomycin (1000 μg/mL) was used as a positive control.

### Antifungal activity of the Cu-particles

The activity of Cu-particles and CuSO_4_ was measured against *Aspergillus niger, Aspergillus flavus, Penicillium chrysogenum, Fusarium solani* and *Alternaria solani*. These strains were provided by the City of Science & Technology, Egypt. Each fungal strain was grown on potato dextrose agar (PDA) slant and incubated at 25 ± 2 °C for 5 days. Three milliliter sterile distilled water was added to each fungal slant and the fungal spore concentration was determined by haemocytometer. One hundred milliliter PDA containing various Cu-particles or CuSO_4_ levels (50, 100, 150, 200 and 250 μg/mL) was inoculated with the fungal spore suspensions (10^6^ spore/mL). After incubation at 25 ± 2 °C for 5 days the cultures were filtered through pre-weighed Whatman No.1 filter paper and the filter paper with fungal biomass was dried at 70 °C until constant weight. At the same time antifungal activity of Cu-particles and CuSO_4_ was evaluated using fungal growth inhibition assay as described by Fiori et al., [29] with some modification. The Cu-particles and CuSO_4_ were mixed with molten PDA to provide desired concentration (200 μL/mL) and 8 mm diameter disc of each fungal strain was added to the center of PDA plates. After incubation at 25 ± 2 °C for 72 h, colony diameter was measured. Nystatin (300 μg/mL) was used as a positive control.

### Treatment of stone blocks with Cu-particles based on polymers

Cu-particles were combined with the consolidation polymers at the concentration 150 μg/mL. The functionalized polymers were used to coat the external surfaces of stone blocks and were left 7 days at room temperature for complete drying.

### Antimicrobial activity of the treated stones

The antibacterial activity of the treated stones was assayed according to Essa and Khallaf [19]. One surface of the coated stones was submerged in the bacterial culture (1.0 × 10^6^ cell/mL) for 2 h then they were incubated at 30 °C for 24 h. After that the treated stones were dipped into 10 mL 0.85% NaCl solution for 1 h. One milliliter of the washing solution was diluted 100 times and 0.1 mL of diluted solutions was plated on NA. After incubation at 30 °C for 24 h the bacterial colonies were counted. Untreated stone samples were used as reference. The experiment was repeated three times with three replicates for each treatment.

### SEM and EDX of the composite Cu-particles based on polymers

The coated surfaces of the stones were analyzed using scanning electron microscope (JEOL JSM-5410, Japan) meanwhile the chemical analysis of the treated polymers were studied using Electron Dispersive X-ray system connected with the scanning electron microscope.

### Statistical methods

The resulted data were tested by using the ANOVA test for significance. Means were compared by least significant differences (LSD) test at levels *P <0.05* and *P <0.01*. All statistical tests were carried out using SPSS (v. 16.0) software.

## Results

### Antimicrobial activities of the Cu-particles

**Fig. 1:**
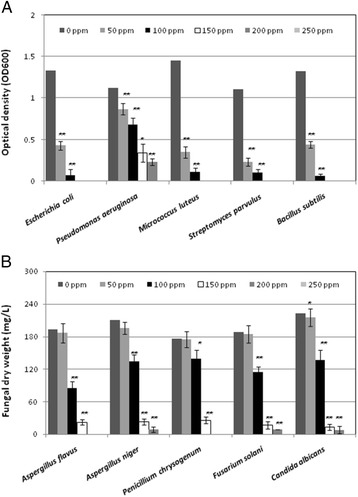
Antimicrobial activity of different concentrations of CuNPs against bacteria (**a**) and fungi (**b**). The bacterial growth was monitored as culture optical density (OD_600_) while the fungal growth was assayed as biomass dry weight. Data are the means of five replication ± standard errors. Means were compared by least significant differences (LSD) test at levels *P* <0.05 and *P* <0.01

**Fig. 2:**
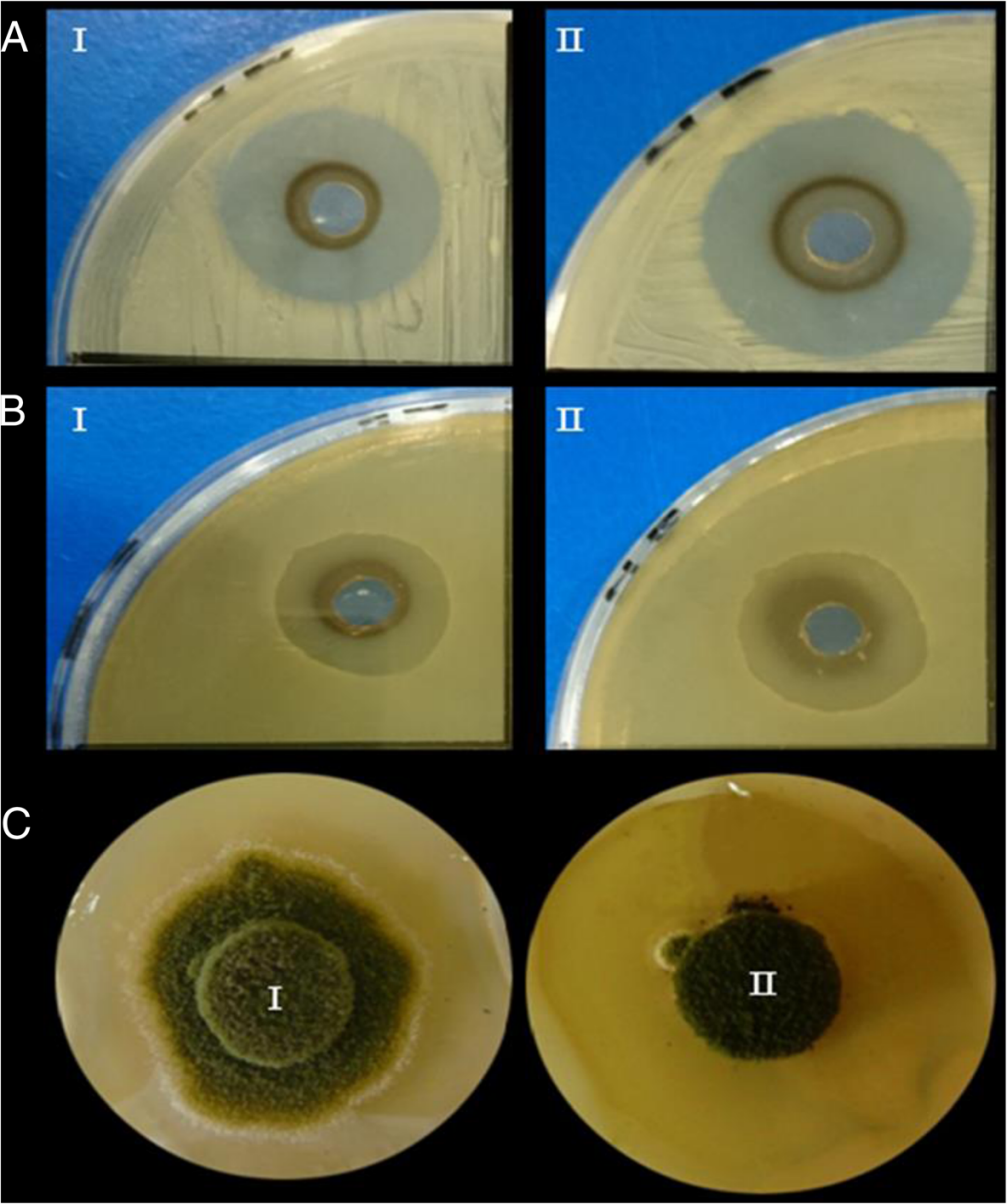
Antimicrobial activity of the different copper forms against (**a**) *Escherichia coli* Z1, (**b**) *Streptomyces parvulus* and (**c**) *Aspergillus flavus* where (*I*) refers to CuSO_4_ and (*II*) refers to CuNPs. Antibacterial activity of Cu-particles and CuSO_4_ was carried out at the concentration 100 μg/mL while antifungal activity was assayed at the concentration 200 μg/mL

As a result of pumping the biogenic volatiles of the bacterial strain *Escherichia coli* Z1 in the copper sulfate solution for short exposure time (60 min), a light blue colloidal solution of copper was obtained. The antibacterial potentiality of the collected Cu-particles was demonstrated at different concentrations. Results in Fig. 1a showed a suppression of the bacterial growth at various levels depending on Cu-particles concentrations. There was no bacterial growth at the concentration 150 μg/mL or above while at 100 μg/mL the percentage of growth reduction reached 94.7 % for *E. coli*, 92.4 % for *M. luteus*, 90.9 % for *S. parvulus*, and 95.5 % for *B. subtilis.* At the same time, *P. aeruginosa* demonstrated a clear tolerance against high concentrations of Cu-particles where the percentage growth inhibition was 69.6 % at 150 μg/mL and 79.5 % at 200 μg/mL. In order to confirm the antibacterial activity of Cu-particles in comparison with untreated copper sulfate, another experiment was conducted where the diameter of the inhibition zones was measured. The obtained results (Table 1 and Fig. 2) showed an increase of the antibacterial activity of Cu-particles compared to CuSO_4_. The maximum inhibition zones were recorded with Cu-particles (150 μg/mL) against *E. coli Z1* (38 mm) and *S. parvulus* (33 mm) while the lowest value was recorded with *P. aeruginosa* (16 mm).

**Table 1:** Antimicrobial activity of Cu-particles and CuSO_4_

Bacterial strains	Inhibition zone (mm)				
	*Escherichia coli*	*Pseudomonas aeruginosa*	*Micrococcus luteus*	*Streptomyces parvulus*	*Bacillus subtilis*
Streptomycin (0.1 mg/mL)	30 ± 1	28 ± 2	25 ± 2	31 ± 1	27 ± 2
CuSO_4_ (100 μg/mL)	31 ± 2**	8 ± 2**	17 ± 1**	21 ± 2*	13 ± 1*
CuSO_4_ (150 μg/mL)	34 ± 1*	9 ± 1**	21 ± 2*	25 ± 1**	18 ± 2**
Cu-particles (100 μg/mL)	37 ± 1**	11 ± 1**	23 ± 1**	24 ± 2*	21 ± 2**
Cu-particles (150 μg/mL)	38 ± 2**	16 ± 2*	29 ± 1**	33 ± 2**	29 ± 1**
Fungal strains	Radial diameter (mm)				
	*Aspergillus niger*	*Aspergillus flavus*	*Penicillium chrysogenum*	*Fusarium solani*	*Alternaria solani*
Nystatin (0.3 mg/mL)	13 ± 1	11 ± 2	16 ± 2	12 ± 2	18 ± 1
Without copper	28 ± 1	31 ± 1	27 ± 1	28 ± 1	33 ± 1
CuSO_4_ (150 μg/mL)	19 ± 2*	17 ± 1**	16 ± 1*	21 ± 1**	20 ± 2*
CuSO_4_ (200 μg/mL)	17 ± 2**	14 ± 1*	13 ± 1**	15 ± 1*	17 ± 2**
Cu-particles (150 μg/mL)	13 ± 1**	11 ± 1**	13 ± 2*	14 ± 1**	15 ± 1**
Cu-particles (200 μg/mL)	11 ± 1*	10 ± 1**	10 ± 2**	10 ± 1**	12 ± 1*

Statistical significance of differences compared to control (without copper): *, significant at *P* < 0.05; **, significant at *P* < 0.01

Agar well diffusion method was used to assay antibacterial activity meanwhile antifungal activity was measured using fungal growth inhibition assay. The values are means of three replicates ± standard error

Regarding the antifungal activity of the Cu-particles, data in Fig. 1b showed a remarkable growth inhibition of *A. flavus, A. niger, P. chrysogenum, F. solani* and *A. solani*. The fungal growth was completely disappeared at the concentration 250 μg/mL while at 200 μg/mL the recorded percentage of the inhibition was 95.7 % for *A. niger*, 95.2 % for *F. solani* and 97.4 % for *A. solani.* At the same time, data in Table 1 and Fig. 2 demonstrated the antifungal activity of Cu-particles in comparison to CuSO_4_. Generally, the antifungal activities of copper were enhanced by increasing their concentration and Cu-particles recorded higher activities than those of CuSO_4_. The maximum growth reduction was recorded at 200 μg/mL of Cu-particles against *A. flavus* (67.7 %) and F. solani (64.3 %) while the lowest growth inhibition was recognized with *A. niger* (60.7 %).

### Antimicrobial activity of the stones treated with Cu/polymer composites

Although elevated concentrations of Cu-particles demonstrated superior antimicrobial potentialities, silicon and acrylic polymers were functionalized with 150 μg/mL of Cu-particles in order to minimize color change of the treated stones. Data in Table 2 showed the antibacterial activities of the treated stones against *E. coli*, *S. parvulus* and *B. subtilis*. The treated sandstone blocks recorded a clear reduction in the percentage of the bacterial cell recovery; 90.1 & 89.1 % for *E. coli*, 95.2 & 92.0 % for *S. parvulus* and 95.3 & 94.8 % for *B. subtilis* with the functionalized silicon and acrylic polymers, respectively. At the same time, the treated limestone blocks demonstrated a clear suppression in the percentage of cell recovery of *E. coli* (93.1 & 86.8 %), *S. parvulus* (93.4 & 95.2 %) and *B. subtilis* (93.1 & 95.0 %) for the functionalized silicon and acrylic polymers, respectively.

**Table 2:** Bacterial cell recovery from sandstone and limestone blocks treated with silicon (S) and acrylic (AC) polymers impregnated with copper nanoparticles

Bacterial strains	Bacterial cell number (x10^4^ CFU/ml)				
	Control	Limestone blocks		Sandstone blocks	
		S/CuNPs	AC/CuNPs	S/CuNPs	AC/CuNPs
*Escherichia coli*	75.8 ± 0.9	5.2 ± 0.3**	9.9 ± 0.4**	7.5 ± 0.6*	8.3 ± 0.3**
*Streptomyces parvulus*	95.3 ± 0.7	6.3 ± 0.5*	4.5 ± 0.2**	4.6 ± 0.4**	7.6 ± 0.6**
*Bacillus subtilis*	84.7 ± 0.6	5.8 ± 0.6**	4.2 ± 0.3**	3.9 ± 0.3**	4.4 ± 0.1**

Statistical significance of differences compared to untreated stone samples: *, significant at *P* < 0.05; **, significant at *P* < 0.01

### Physical and mechanical properties of the treated stones

Results in Table 3 demonstrated a clear improvement in the physical and mechanical properties of the tested stones as a result of the application of the consolidation polymers. Silicon and acrylic polymers showed an increase in the percentage of bulk density, compressive strength and tensile strength of the treated stones. Meanwhile the water absorption capacity and porosity of the treated stones were sharply reduced as a result of using the consolidation polymers.

**Table 3:** Physical and mechanical properties of sandstone and limestone samples treated with the functionalized silicone (S) and acrylic (AC) polymers

Physical and mechanical properties	Sandstone			Limestone		
	Untreated stones	AC/CuNPs	S/CuNPs	Untreated stones	AC/CuNPs	S/CuNPs
Bulk Density (g/cm^3^)	1.6 ± 0.3	1.9 ± 0.4*	1.8 ± 0.2	1.9 ± 0.3*	2.2 ± 0.2**	2.1 ± 0.3*
Water Absorption (%)	19.8 ± 1.9	14.3 ± 1.7*	3.6 ± 0.4**	8.4 ± 1.1**	6.4 ± 0.5**	2.3 ± 0.2**
Porosity (%)	26.3 ± 2.5	18.7 ± 2.2*	4.3 ± 0.3**	15.6 ± 1.5*	9.7 ± 0.8**	4.4 ± 0.4**
Compressive strength (MPa)	19.8 ± 1.6	28.3 ± 1.9**	26.9 ± 2.7**	26.9 ± 2.2**	39.8 ± 2.7**	32.5 ± 1.7**
Tensile Strength (MPa)	3.2 ± 0.6	4.9 ± 0.4**	4.2 ± 0.6	4.3 ± 0.7*	5.3 ± 0.4**	4.9 ± 0.5**

Statistical significance of differences compared to untreated stones: *, significant at *P* < 0.05; **, significant at *P* < 0.01

Data are the means of three replication ± standard errors

### SEM & EDX analysis of the composite Cu-particles based on polymers

**Fig. 3:**
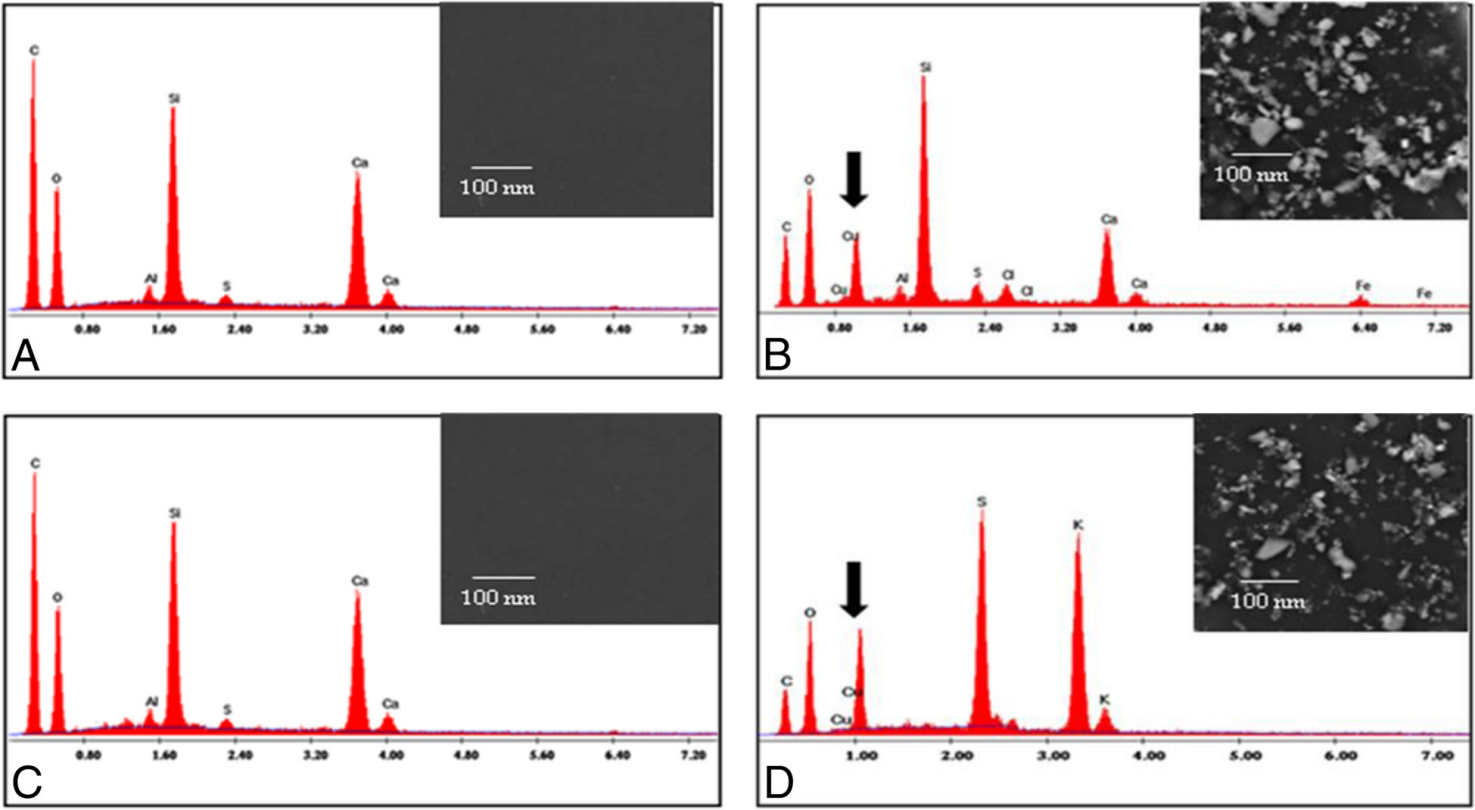
SEM images and EDX analysis of the unfunctionalized silicon polymer (**a**), silicon polymer functionalized with CuNPs (**b**) and unfunctionalized acrylic polymer (**c**), acrylic polymer functionalized with CuNPs (**d**) while *black arrows* indicate the elemental copper

The current study Fig. 3 showed the analysis of the treated and untreated polymers with composite copper structures by scanning electron microscope. In case of the functionalized polymers, tiny particles (10–50 nm in diameter) were identified while these particles were absent in the un-functionalized polymers. At the same time, the EDX analysis of these minute structures showed the presence of the elemental copper in the treated silicon and acrylic polymers in addition to oxygen, carbon, sulfur, silicon, potassium, chloride and calcium elements. Furthermore, the EDX analysis of the un-functionalized polymers showed the presence of carbon, silicon and oxygen elements with the silicon polymer while the peaks of carbon, oxygen, silicon, chloride, calcium and aluminum peaks were recognized in case of the acrylic polymer.

## Discussion

In our previous study [27], the capability of some bacterial strains for the precipitation of various metal ions out of their solutions was recorded using the culture biogas. In the present work, Cu-particles were prepared biologically via exposing the copper ions to the biogenic volatiles released during the aerobic growth of *Escherichia coli* Z1. One of the main constitutes of these gases is ammonia that is responsible for the transformation of copper ions into nitrogen-based copper particles [30]. The existence of ammonia in bacterial biogas is mainly attributed to the catabolic reactions of some organic matter [31].

At minor concentration of ammonia, the aqueous copper sulfate solution produces copper hydroxide while high ammonia levels induces the formation of diammine copper (II) complex [Cu(NH_3_)_2_]^+^ [27]. In fact, the alteration of the copper ions into colloidal copper particles is correlated with the exposure time. At short exposure time, minute copper particles (10–50 nm) were formed as showed in the SEM analysis. The EDX analysis of the copper structures clarified the existence of sulfur that could be attributed to the incidence of volatile organothiol compounds in the bacterial biogas [32].

This study clarified a marked antimicrobial efficacy of CuNPs against various bacterial and fungal species. The biocidal activity of CuNPs could be attributed to the effect of the CuNPs and/or the copper ions discharged from CuNPs. Because of the great surface area of the nanoparticles, it could be tightly adsorbed onto the surface of the microbial cells resulting in; i) disruption of cell permeability and release of integral components [33], ii) denaturing of some functional biomolecules [10, 13], iii) induction of oxidative damage to the microbial cells. However, some studies have reported that the liberated Cu^2+^ is the motivating force behind the antimicrobial properties of polymers containing Cu-nanocomposites [2, 34]. At the same time, the discharged copper ions might be moved inside the microbial cells or attached to their outer surfaces resulting in cell apoptosis via protein denaturation and disruption of cell membrane [35, 36]. Obviously, nonspecific mode of action of Cu^2+^ or CuNPs against bacteria and fungi makes them perfect antimicrobial agents with low possibility of developing microbial resistance [4, 33].

In the current study, the silicon and acrylic polymers that were loaded with the copper nanoparticles showed a positive influence on the treated stones through suppressing the growth of tested bacterial strains at various levels. At the same time, the antimicrobial activity of the biosynthesized CuNPs was not changed by merging with consolidation polymers. These results are in agreement with our preceding study [19] that revealed the protection of some archeological stone against microbial colonization via the application of consolidation polymers/AgNPs composites onto their surfaces. Similarly, Pinna et al. [37] clarified a superior protective behavior against the microbial colonization on stones via treating them with consolidants loaded with copper nanoparticles.

In addition to the antimicrobial task of the functionalized polymers, they demonstrated an apparent perfection in the physical and mechanical properties of the treated stones. The consolidation polymers especially silicone polymer decreased the level of water absorption and porosity of stones through the formation of a protective layer. This layer is formed due to the penetration of the polymer molecules into voids and pores of the stone matrix. Moreover, the mechanical measurements indicated that both types of polymers increased the compressive strength which reflects the importance of using these polymers in the consolidation processes of the limestone monuments. These results are in harmony with Ahmed [38] who recorded a marked improvement in the physico-mechanical behavior of limestone samples after treating them with some synthetic polymers. Also, Khallaf et al. [39] showed an increase in bulk density and decrease in porosity as well as increase in compressive strength of the monuments made of sandstone and limestone after treating them with some organic polymers.

## Conclusion

Copper has strong biocidal activity with non-specific mode of action against microbial cells that make it ideal antimicrobial agent. CuNPs were prepared through novel bioprocess that utilizes volatile metabolites of *Escherichia coli* to aggregate Cu ions into nanometal structures away from the bacterial cells. This bioprocess is inexpensive and eco-friendly. Besides, uncontaminated bacterial biomass could be used safely in different applications. The incorporation of CuNPs into polymer matrix produced nanocopper composites with remarkable antimicrobial capability. The functionalized consolidation polymers could be used not only to inhibit the microbial growth on the surfaces of historical stones but also to improve physical and mechanical properties of the treated stones. Additional research is required to evaluate the application of consolidation polymers loaded with nanoparticles of copper in situ treatment.
